# Successful Curriculum Change in Health Management and Leadership Studies for the Specialist Training Programs in Medicine in Finland

**DOI:** 10.3389/fpubh.2018.00271

**Published:** 2018-09-21

**Authors:** Heli M. Parviainen, Heli Halava, Esa V. J. Leinonen, Elise Kosunen, Pasi-Heikki Rannisto

**Affiliations:** ^1^Department of Health Sciences, Faculty of Social Sciences, University of Tampere, Tampere, Finland; ^2^Faculty of Medicine, University of Turku, Turku, Finland; ^3^Faculty of Medicine and Life Sciences, University of Tampere, Tampere, Finland

**Keywords:** management-healthcare, specialist training in medicine, management education and development, leadership and physicians, medical specialist training and management

## Abstract

In Finland, the specialization programs in Medicine and Dentistry can be undertaken at all five university medical faculties in 50 specialization programs and in five programs for Dentistry. The specialist training requires 5 or 6 years (300–360 ECTS credits) of medical practice including 9 months of service in primary health care centers, theoretical substance specific education, management studies, and passing a national written exam. The renovation of the national curriculum for the specialization programs was implemented, first in 2008 and officially in August 2009, when theoretical multi-professional social, health management and leadership studies (10–30 ECTS credits) were added to the curriculum. According to European Credit Transfer and Accumulation System (ECTS), 1 ECTS credit (henceforth, simply “ECTS”) means 27–30 h of academic work^1^ National guidelines for the multi-professional leadership training include the basics of organizational management and leadership, the social and healthcare system, human resources (HR) management, leadership interaction and organizational communication, healthcare economy, legislation (HR) and data management. Each medical faculty has implemented management studies autonomously but according to national guidelines. This paper will describe how the compulsory management studies (10 ECTS) have been executed at the Universities of Tampere and Turku. In Tampere, the 10 ECTS management studies follow a flexible design of six academic modules. Versatile modern teaching methods such as technology-assisted and student orientated learning are used. Advanced supplementary management studies (20 ECTS) are also available. In Turku, the 10 ECTS studies consist of academic lectures, portfolio and project work. Attendees select contact studies (4–6 ECTS) from yearly available 20 ECTS and proceed at their own pace. Portfolio and project comprise 2–5 ECTS each. The renovation of medical specializing physicians' management and leadership education has been a successful reform. It has been observed that positive attitudes and interest toward management overall are increasing among younger doctors. In addition, management and leadership education will presumably facilitate medical doctors' work as managers also. Continuous development of medical doctors' management and leadership education for physicians and dentists is needed while the changing and complex healthcare environment requires both professional and leadership expertise.

## Introduction

Continuous development of physicians' management and leadership education is needed while the changing and complex healthcare environment requires not only professional expertise but also expertise in comprehensive and collaborative leadership (1–3). Professionals in health care are working more and more often in multi-professional teams. Hence, it is no longer possible to concentrate on medical expertise only and to ignore management and leadership.

Meanwhile, critical observations on insufficient skills for management and leadership among physicians have emerged (1, 3, 4). Among physicians, difficulties have been observed in finding a balance between leadership and clinical work (5). The transition to become a “hybrid,” clinical leader, often also requires leaders to move outside their comfort zone (2, 6, 7). Physicians are expected to take responsibility for the management of financial and human resources in health care, especially in hospitals (1) but often without previous management training or mentorship (8). In health care, it is also common that physicians have been appointed as managers according to their clinical expertise, scientific qualifications and/or seniority (9, 10). Expertise in a certain profession does not automatically also qualify one to practice management and leadership (11). Core management and leadership competencies have to—and can—be formally educated (2).

Earlier in Finland, since 1960's, education for health care administration was mainly provided to senior physicians as further education or short courses. Twenty hours course on administration in health care was included in medical specialization training in 1978 and the requirement was valid for more than 30 years (12). At the same time, many other healthcare professionals were required to undertake an increased amount of leadership training as a part of their education (13–15). However, the very short course did not satisfy most physicians because basic knowledge of management and leadership could not be achieved (16, 17). In addition to education on clinical skills, the introduction of management competencies should start early in the career to develop a seamless educational continuum for future health care leaders and to ensure that future specialists are capable “team-players,” e.g., communicating and giving constructive criticism and making decisions together (2). Two earlier Finnish surveys (Physician 2008 and Physician 2013) showed that gynecologists, pediatricians and neurologists, in addition to senior physicians with a managerial position, were more often dissatisfied with management, leadership and administration education included in their specialist training, while more often satisfied were GPs and physicians in surgery specialties. No differences were observed between genders, however most of the respondents in gynecology and pediatrics were female physicians, while surgery specialties were male-dominated (17).

Thus, it was obvious, that the curriculum of Medical specialist training needed a renovation. Finally, in the report of the Ministry of Social Affairs and Health in 2007 it was suggested that multiprofessional management and leadership education of minimum of 10 ECTS should be included in all specialization programs (16, 18).

During the renovation of the curriculum in 2009, Universities of Oulu, Eastern Finland, Tampere and Turku included 10 ECTS theoretical management and leadership studies in specialist training, whereas University of Helsinki had 30 ECTS compulsory for all specializing physicians. However, also in the University of Helsinki the extent of compulsory management studies included in medical specialization was changed to 10 ECTS starting 1.1.2018.

A national guideline for the management and leadership studies incorporated into curriculum of the medical specialist degree was accepted in all five medical faculties in Finland. However, each medical faculty has implemented management studies autonomously but according to the national guidelines.

This paper will describe how these studies (10 ECTS) have been implemented in the Universities of Tampere and Turku and what specializing physicians think about management education.

### The specialist training in medicine in finland

**Figure 1:**
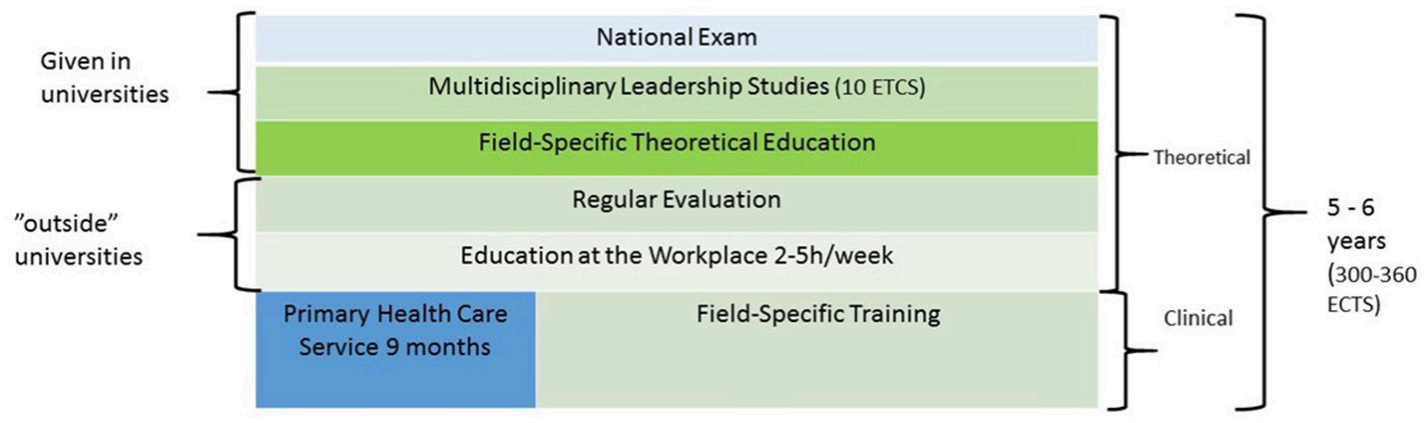
The structure of specialist training in medicine in Finland.

In Finland, the specialist training in medicine is classified as specialized postgraduate degree. It can be undertaken at all five medical faculties in Finland. To complete the specialist degree, 5 or 6 years (300–360 ECTS) of medical practice is required, including 9 months of service in primary health care centers, theoretical courses, management studies, and passing a national written exam. At least half of the total training time must be completed outside the university hospital, except for the Programs mentioned in Figure 1 (Decree on education of specialist physicians and dentists 56/2015 § 6^2^).

The universities of European Higher Education Area (EHEA)^3^ use European Credit Transfer and Accumulation System (ECTS) to describe dimensioning of an educational program and aimed learning outcomes. One ECTS means 27–30 h of academic work (ENIC-NARIC^4^).

The curricula of all specialization programs for physicians and dentists in the Universities of Tampere and Turku include 10 ECTS of compulsory, theoretical, multi-professional, social and health management studies. These studies are offered according to the national curriculum consisting of the basics of organizational management and leadership, the social and healthcare system, human resources (HR) management, leadership interaction and organizational communication, healthcare economy, HR legislation and data management. In addition to compulsory management studies, specializing physicians are offered an option to undertake an additional, voluntary 20 ECTS of studies in management and leadership.

For the part of management and leadership studies, the specialization training in dentistry is congruent with specialization in medicine including the same 10 ECTS of management studies.

### Health management studies in the university of tampere

**Figure 2:**
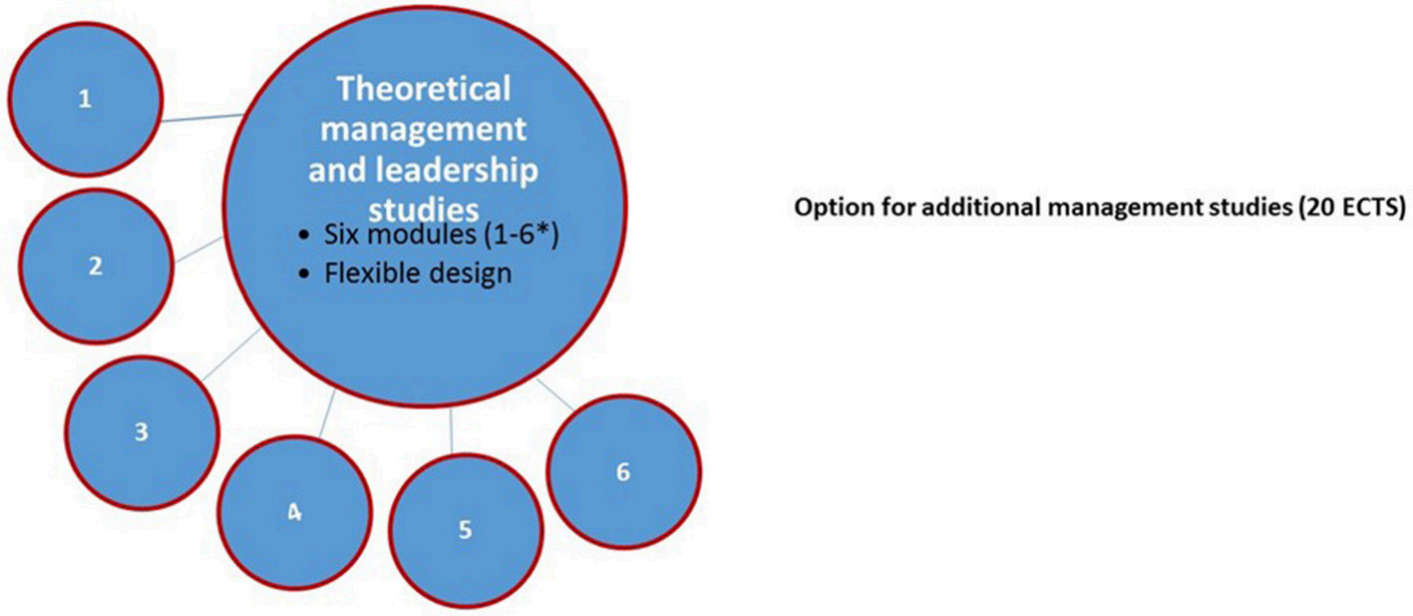
Compulsory management and leadership studies (10 ECTS) for specializing physicians in the University of Tampere. ^*^ (1) Organizational management and leadership [(1 ECTS) e.g., management of a health care organization; customer-oriented approach in health care; managerial work in health care]; (2) Social and healthcare system [(2 ECTS) e.g., history and future of the social and health care and welfare system in Finland; social justice and quality of life; reform of the social and healthcare system in Finland]; (3) Human resources (HR) management [(2 ECTS) e.g., managing professionals; management and leadership of working teams; interpersonal workplace skills; self-management, occupational health of physicians]; (4) Leadership interaction and organizational communication [(2 ECTS) e.g., interaction in management; public relations; communicating in social media as a health care professional; workplace communication]; (5) Healthcare economy [(2 ECTS) e.g., why and how (in practice) to pay attention to economy and cost-effectiveness as a manager in health care]; (6) HR legislation and data management [(1 ECTS) e.g., legislation related to human resources management; legislation of patient safety and management; legislation of public procurements; data management in health care organizations; digitalization of health care services].

In the University of Tampere, the 10 ECTS management studies follow a flexible design of six separate modules according to the national guidelines. Students are free to plan the timing of the study modules and participation during their specialist training (Figure 2).

Pedagogical solutions used for each subject 1-day-module include academic lectures on the theoretical bases, the integration of theories into health care practices, as well as student-inspired workshops based on preliminary orientating reading materials assisted with web-based solutions (e.g., Padlet, Answer Garden etc.) utilizing BYOD-pedagogy (Bring Your Own Device). Furthermore, the online education network Moodle, as well as, gamification [JOPE serious virtual game (19)] has been used, e.g., for completing web-based assignments to deepen trainees' understanding on academic lecture topics.

The Department of Health Sciences in The Faculty of Social Sciences organizes both compulsory and supplementary studies in co-operation with The Faculty of Medicine and Life Sciences with financial support from the Hospital Districts of Kanta-Häme, Päijät-Häme (2008–17), Pirkanmaa, South Ostrobothnia and Vaasa (2008–14). The contact education has taken place in the central hospitals of Hämeenlinna, Lahti, Seinäjoki and Vaasa in addition to the University of Tampere. This has enabled specializing physicians to participate in management studies also close to their learning through service period workplaces.

The constant feedback after every module was first collected with paper forms, but lately as open feedback using a virtual Padlet “wall.” Nowadays, the feedback has been very positive: in particular the flexibility of the education template, relevance of the subjects included in the compulsory management and leadership studies, possibility to have discussions with lecturers and in small groups with colleague trainees and the offering of education near to the students. Discussions about why medical trainees must use their time in studies like management instead of clinical training no longer appear like they used to in the very first years after the curriculum renovation, when the extent of management and leadership studies increased from 20 h to 10 ECTS.

Students are also provided an additional option for advanced and supplementary management studies (20 ECTS). For these modules, students can choose team-based tutored eLearning assignments applied to health care practices (5 × 2 ECTS) in subjects such as managing professionals, ethics and HR, project management, and understanding key financial indicators related to the economics of health care organization. However, to every eLearning assignment also face-to-face (or Skype) kick off or summary seminar is included. Also, modern book exams (5 × 2 ECTS) conducted as face-to-face sessions in teams using flipped classroom pedagogy on current topics in health care management and leadership are offered. In addition, recognition of prior learning (RPL) is possible when it comes to, e.g., earlier management studies, research or manager experience. At the same time, while management and leadership substance are studied, communication and teamwork skills are also practiced. Upon request, the students receive a separate certificate of their 30 ECTS management and leadership studies. All completed studies are marked in the student's study register.

Even though the additional 20 ECTS of courses can be done mainly as distance learning assignments with flexible timing, only 4–5% of all specializing physicians have completed the 30 ECTS management and leadership studies.

### Health management studies in the university of turku

**Figure 3:**
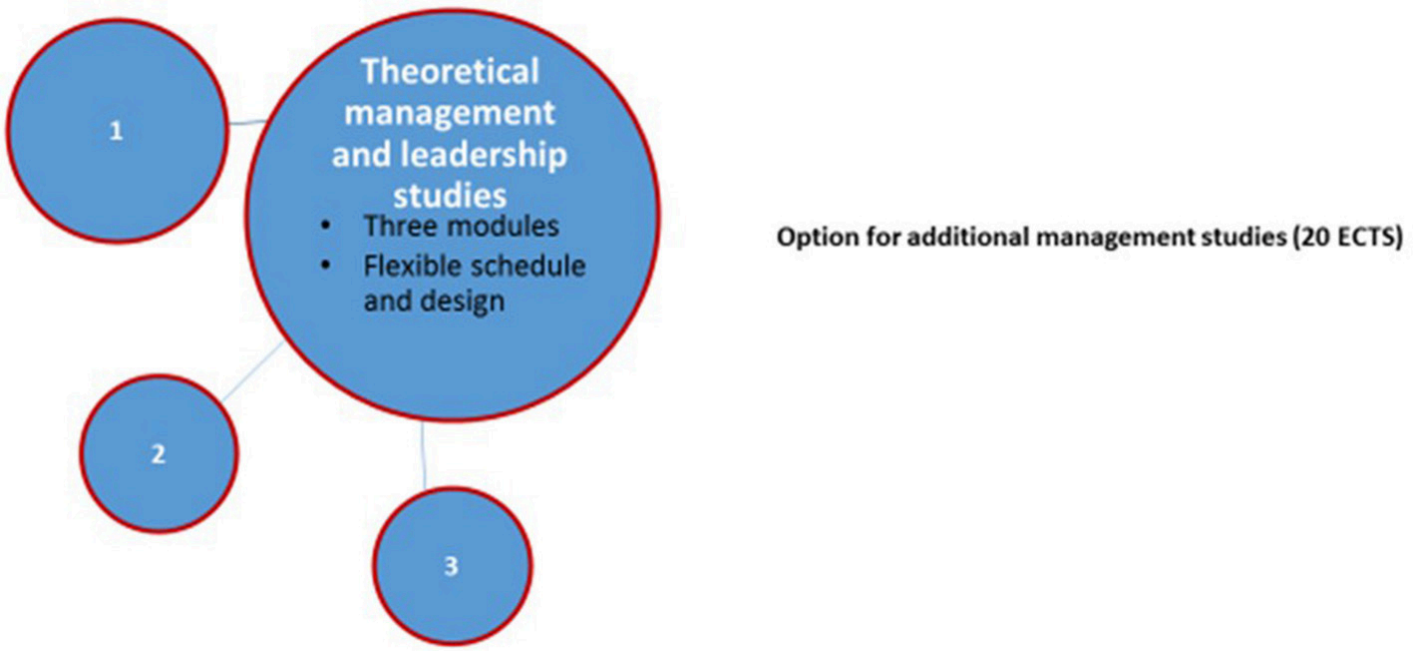
Compulsory management and leadership studies (10 ECTS) for specializing physicians in University of Turku. (1) Contact studies (in total 3-6 ECTS) in relation to organizational management and leadership, social and healthcare system, human resources management, leadership interaction and organizational communication, healthcare economy and law. (2) Portfolio (minimum 2 ECTS and duration for 1 year). (3) Project work (minimum 2 ECTS).

In the University of Turku, management studies consist of contact studies, portfolio and project work. Attendees select contact studies (3–6 ECTS) from yearly available 20 ECTS and proceed at their own pace. The Faculty of Medicine has organized these studies with financial support of The Hospital District of Southwest Finland and in co-operation with faculties of Law and Social Sciences and School of Economics to gain a multi-professional perspective. Portfolio and project comprise 2–5 ECTS each and it is up to the attendee's discretion to decide on the proportion of these three sections (Figure 3).

Contact studies are arranged as academic lectures on the theoretical bases, including the integration of theories into workshops and conversations to confirm communication and teamwork skills. An additional significance of contact studies among specializing physicians is to create networks where they can share experiences. Also, preliminary orientating reading materials are provided and the online education network Moodle is utilized and, exercises, assignments and exams may be included. The contact studies have taken place in Turku in addition to online video provided, to enable trainees to participate nearby their workplaces.

The majority on contact studies consist of 2 ECTS courses but also 1 ECTS courses exist. The topics of the courses adhere to a 2-year-rotation, which has been revised on demand. The constant feedback is collected after each course and it has been mostly laudatory. The attendees also appreciate the opportunity to choose the courses according to their own schedule and interest and, during courses to meet trainees of other specialization fields as well as specialists.

The main objective of the project work is to connect leadership studies to clinical work. A specializing physician is supposed to formulate, together with her/his supervisor, an administrative project that will benefit both the workplace and their employees or patients. The combined experience of quality improvement and education makes these projects a good initiation into further managerial roles (20). Portfolio work, meanwhile, is supposed to enhance the trainee's ability to take responsibility as a team leader, to support professional growth and to highlight targets for development. It may include, for example, a SWOT analysis or curriculum vitae. Another tool for learning through project and portfolio is mentoring: every specializing physician decides on a supervisor, who guides the trainee through project work and portfolio as a senior colleague. The main objectives of mentoring are personal and professional development of the mentee with some benefit for the mentor. The mentor is acting as a guide to the mentee in a non-formal and non-structured way.

In Turku, students are also provided optional management studies (extra 20 ECTS). However, only 2–5% of specializing physicians have completed the 30 ECTS management and leadership studies during their specialist training.

### The volume of medical specialization trainees and graduated medical specialists

During the years 2009–2017, the number of specializing physicians, as well as the number of graduated medical specialists, has shown a slightly increasing trend in the University of Tampere (Table 1). Also, in the University of Turku, the number of graduated medical specialists has shown a moderate increase whereas the number of trainees starting specialist training has increased considerably (Table 1).

**Table 1:** Number of specializing physicians and graduated medical specialists in Universities of Tampere and Turku in 2009–2017.

**Year**	**Specializing physicians—started**		**Graduated medical specialists**	
	**Tampere**	**Turku**	**Tampere**	**Turku**
2009	195	62	119	83
2010	185	35	118	83
2011	277	59	106	77
2012	276	63	123	93
2013	282	85	150	91
2014	302	151	129	88
2015	237	150	127	91
2016	254	130	131	81
2017	244	123	135	100
2009–2017	2,252	858	1,138	787

Table 2 shows the increasing number of participants to compulsory management and leadership studies (10 ECTS). This is in accordance to the number of initiators of specializing physicians. In Tampere, the number of participants has been calculated according to the number of attendees in each module which means that one trainee may have participated in one or more modules during one's specialist training. In Turku, a trainee may participate in three courses, at the most, during the 5 or 6-year specialization program.

**Table 2:** Number of participants in compulsory management and leadership studies (10 ECTS) in 2009–2017.

	**Participants**	
	**Tampere^*a*^**	**Turku^*b*^**
2009	396	na
2010	555	70
2011	543	117
2012	799	171
2013	905	234
2014	984	257
2015	1,053	314
2016	1,054	277
2017	1,141	329

a * One trainee may participate in one or more (maximum 6) modules during the 5 or 6 years of the program*.

b * One trainee may participate in one or maximum of 3 courses during the 5 or 6 years of the program*.

The annual variation may be due to a recent change in the student selection procedure (specializing students will be selected, whereas before they could simply sign up). A small proportion of the increase may also be due to the number of applicants to the new and attractive specialty of acute medicine, which some of the specialized/specializing doctors in other fields have also specialize in.

### Feedback related to the extent of management and leadership education among medical trainees and trainers

After the 2008 renovation of medical specialization program, especially younger physicians reported more often satisfaction with their management and leadership education and competences compared to their senior colleagues (17). Hence, the renovation of doctors' management and leadership education has been a successful reform.

In spring 2017, Halava (16) conducted a survey among specializing physicians and their trainers to study opinions on the appropriate extent of management and leadership education incorporated into the specialization program in the University of Turku. Among trainees, 82% reported the extent of 10 ECTS management studies to be appropriate. Also, among trainers the result (93%) was similar to trainees (16). The results of Turku are in accordance with the results of the Physician 2013 survey: half of young trainees were satisfied with the current extent (10 ECTS) of management education (17).

In Tampere, trainees have been asked to give structured feedback (eForm) also after completing the compulsory management and leadership 10 ECTS program. For the feedback, a Likert-type scale (1 = completely disagree, 5 = completely agree) is used to assess the importance of management studies, structure and execution of the program, availability of information, and the benefit of the management and leadership studies to practice. Giving feedback is not compulsory, and so only a small number of trainees have answered during 2010–2017. The feedback (mean of scores 1–5) concerning completed compulsory studies in the management and leadership 10 ECTS program in 2010–2017 in Tampere can be summarized as follows:

- Management and leadership education is important to medical specialization trainees (4.7./5)
- The structure of the compulsory management and leadership studies 10 ECTS is fitting for the purpose (4.5/5)
- Information regarding the compulsory management and leadership studies 10 ECTS was easily available (4.5/5)
- The management and leadership studies conducted are beneficial to practice (4.5/5)

Hence, the power of the quantitative results (mean of all the responses in 2008–2017) presented is not strong, but in accordance with the qualitative feedback collected after each module.

Both in Tampere and Turku, the steering group of medical specialization management and leadership, the deans, as well as the financing organizations, are informed annually regarding each academic year's implementation. The report includes also the results of collected feedback. In addition, trainees' feedback and ideas have been taken into account for the continuous development of the management and leadership education program.

## Discussion

Although not every doctor will work as a manager, it is also important to have good workplace skills as a member of a professional team. Management and leadership education will presumably make it easier for doctors to also work as managers. Additionally, it appears that positive attitudes toward management overall are increasing among younger doctors (16, 17). In Finland, positive attitudes toward developing specializing trainees' management and leadership skills can also be observed in the employers' contribution to the funding of management education.

According to the feedback received, students consider compulsory studies (10 ECTS) as necessary and useful for the profession and work of a medical specialist. Most younger specializing physicians, as well as their trainers, reported the extent of 10 ECTS management and leadership studies to be appropriate (16, 17).

However, of all specializing physicians, only 2–5% have completed the 30 ECTS management and leadership studies during their specialist training in both Tampere and Turku. According to discussions with specializing physicians, this is partly due to a lack of time required to complete the extra 20 ECTS management studies in addition to medical courses, but also partly due to a lack of interest to be a “hybrid” manager and physician. Most physician leaders choose to continue with their clinical practice (5). Clinicians may feel powerless for being responsible for organizational issues without the time, support or budget to improve the situation, and it may be difficult to control the workload (4, 21–23). A further barrier to physicians is that leadership training rarely affords opportunities to engage in strategy making in hospital or health care centers (24). However, according to Veronesi et al. (25), the representation of clinicians in management would help to improve the performance of a health care organization, e.g., in hospital-level outputs and outcomes (25). In Finland, it is often expected that a medical specialist will also serve as a team leader, or that a GP will take care of the management of a primary health care center in addition to her/his clinical duties. In hospital districts around both Tampere and Turku, employees in the health care organization are also provided further education of various extents in management and leadership by their employers.

Informal and tacit performance of management and leadership, as traditionally conducted in professional organizations such as hospitals, need to be renovated into more explicit and structured procedures (24). In Finland, recent remarkable reforms of the social and health care system, as well as rapid changes in society and in (health) technology also challenge execution of management and leadership to change in health care organizations and among professionals. Health care professionals have to pay more attention to cost efficiency, quality and safety of health care performance, expectations to new service design, to changing mode of their work and communication as members in multi-professional teams, in addition to patients' increasing expectations and demands as consumers of health care services (6, 16, 17, 24). As de Bruijn (11) has stated, expertise in a certain profession rarely qualifies one to also practice management and leadership (11), but it is possible to learn (2). All the specializing physicians who have completed the 30 ECTS management and leadership studies, in both Tampere and Turku, have been extremely motivated to officiate as managers, as well as ready to start to develop management and leadership in health care organizations in the future.

Notably, the increase in the proportion of management and leadership education incorporated into specialization programs since 2008 has developed young physicians' awareness of the markers of good medical leadership, as well as their criticism of managing professionals in health care. Among physicians in specialist training, a good medical leader was emphasized to have good interpersonal and communication skills (good interpersonal skills, discusses and shares, easy to approach, available), leadership skills (supports and guides employees, knows employees' work, respects and values employees, supports professionalism, is fair and just) in addition to good managing expertise (clinical know-how, future envisioning, sharing information, good networker) (26).

To become a health care leader today, long-term, comprehensive and interdisciplinary management and leadership training is needed. Early career management programs for medical students may also be valuable as a basis for further health care leadership training and development. Workplace skills, such as interactive communication and teamwork, economical awareness, as well as a broader understanding of the environment where health care organizations are acting, are basic components required of a health care leader. Continuous development and research of medical doctors' management and leadership education is needed, while the changing, complex healthcare environment additionally requires professional expertise and strong, multifaceted expertise in management and leadership.

## Conclusions

The renovation of doctors' management and leadership education has been a successful reform. However, continuous development of medical doctors' management and leadership education is needed because of the changing healthcare environment which requires managers with both professional and leadership expertise. Although not every doctor will work as a manager, it is also important to have good workplace skills as a member of multi-professional teams.

In addition to education on clinical skills, the introduction of management competencies should be started early in the career to ensure that future specialists are capable team-players. Early career management programs for medical students may also be valuable as a foundation for further health care leadership training and development. Modernization of management and leadership in health care may only be possible if the professionals in this field are offered further education to achieve professional skills also in management and leadership.

## Author contributions

HP is the main and corresponding author. HH is the author of the parts of the article especially regarding the University of Turku. EL, EK, and P-HR provided comments for the article throughout the writing process.

### Conflict of interest statement

The authors declare that the research was conducted in the absence of any commercial or financial relationships that could be construed as a potential conflict of interest.

## Footnotes

^2^https://www.finlex.fi/fi/laki/alkup/2015/20150056.

^3^European Higher Education Area (EHEA) is based on the Bologna Declaration (1999) including 48 European countries (*Statement of the Fifth Bologna Policy Forum* http://media.ehea.info/file/2018_Paris/36/8/BPFStatement_with_Annex_958368.pdf) http://www.ehea.info/pid34247/how-does-the-bologna-process-work.html. The main goal of academic institutions and stakeholders of the EHEA countries is to increase staff and student mobility and to facilitate employability (http://www.ehea.info/).

^4^http://www.enic-naric.net/ects---european-credit-transfer-and-accumulation-system.aspx
